# Pathological Fracture of Thoracic Spine Aneurysmal Bone Cyst: A Case With Successful Outcome Following Debulking, Posterior Instrumentation, and Allogenic Bone Impaction Grafting

**DOI:** 10.7759/cureus.95550

**Published:** 2025-10-27

**Authors:** Mun Han Ho, Muhamad Zharif Nor Asikin, Mohamad Fauzlie Yusof

**Affiliations:** 1 Orthopaedics and Traumatology, Hospital Melaka, Melaka, MYS

**Keywords:** aneurysmal bone cyst, bone grafting, bone impaction, debulking, pathological fracture, posterior instrumentation, spine, thoracic

## Abstract

Aneurysmal bone cysts (ABCs) are benign, osteolytic lesions characterised by blood-filled spaces separated by fibrous septa. Although benign, ABCs exhibit locally aggressive behaviour, leading to significant bone destruction. These cysts primarily affect children and young adults, often occurring in the metaphysis of long bones and the spine. Spinal ABCs are particularly concerning due to the potential for neurological deficits caused by spinal cord compression. The pathogenesis of ABCs remains under investigation, with evidence suggesting both a vascular origin and genetic abnormalities involving the USP6 gene. Diagnosis requires a multi-modal approach combining clinical assessment, imaging, and histology.

Management strategies for ABCs range from surgical curettage and en bloc resection to minimally invasive techniques such as percutaneous sclerotherapy. Adjuvant therapies, including bone cement and Denosumab, are employed to reduce recurrence. In cases involving pathological fractures or neurological impairment, surgical intervention is often necessary. Despite these advances, recurrence remains a concern, particularly in spinal lesions.

This case study discusses a 56-year-old male with a T11 pathological fracture secondary to a spinal ABC. He underwent neural decompression, debulking, and posterior spinal fusion, achieving favourable outcomes. The case highlights the challenges of treating ABCs, the importance of early diagnosis, and the role of multi-disciplinary management in preventing recurrence and ensuring long-term stability.

## Introduction

Aneurysmal bone cysts (ABCs) are non-malignant, osteolytic bone lesions characterised by blood-filled cavities separated by fibrous septa containing multinucleated giant cells and areas of reactive bone formation. Despite their benign status, ABCs are locally aggressive and can lead to significant bone destruction and potential deformities. They constitute about 1% of all primary bone tumours and predominantly affect children and young adults, typically presenting within the first two decades of life. Common sites of occurrence include the metaphyseal regions of long bones like the femur, tibia, and humerus, as well as the vertebral column. Approximately one-third of these cysts originate in the spine, accounting for around 15% of all primary spinal tumours. Clinically, ABCs manifest as localised pain, swelling, and occasionally pathological fractures. Neurological deficits may develop when spinal cysts compress neural elements. Pathological fractures occur in about 8% of ABC cases [1]. Spinal ABCs, while rare, are histologically benign tumours with aggressive behaviour, capable of causing bone and soft-tissue destruction, particularly affecting neural structures. Treatment strategies for ABCs are diverse, ranging from surgical curettage and bone grafting to minimally invasive techniques such as percutaneous sclerotherapy and radiofrequency ablation. Adjuvant therapies, including the use of phenol, liquid nitrogen, or bone cement, and newer pharmacological treatments such as Denosumab, have shown promise in reducing recurrence rates. Despite generally favourable outcomes, the propensity for recurrence necessitates vigilant long-term follow-up.

We describe a successful case involving a 56-year-old man with a T11 pathological fracture secondary to ABC. He underwent neural decompression, debulking, and posterior spinal instrumentation and fusion from T9 to L1, with cage insertion at T11. At the six-month follow-up, the patient remained symptom-free and ambulatory without assistance. He has consented to the publication of his case data.

## Case presentation

A 56-year-old Malay man presented with a two-month history of progressive lower back pain prior to attending the orthopaedic clinic. At the initial consultation, his pain intensity was recorded as seven out of 10 on the numeric pain rating scale. Over the course of this period, the pain had progressively worsened, resulting in functional limitations that interfered with his occupation as an electrical technician. He reported difficulty climbing ladders and lifting objects due to the persistent discomfort. He did not report any weakness or numbness. On examination, there was palpable midline tenderness over the lower thoracic and upper lumbar regions. The neurological examination showed intact bilateral lower limb power and sensation, with normal tone and reflexes.

Thoracic X-ray revealed a T11 compression fracture. Due to the persistent pain and findings on the initial plain radiograph indicating a compression fracture, further assessment with magnetic resonance imaging (MRI) of the spine was performed. MRI imaging showed a multiloculated lesion with fluid-fluid levels at the T11 vertebral body, with paraspinal swelling causing cord compression.

He underwent neural decompression, debulking, and posterior spinal instrumentation and fusion from T9 to L1 with cage insertion at T11. Following the induction of general anaesthesia, the patient was positioned in the prone position. A midline incision was made over the posterior thoracolumbar region. Intraoperatively, multiple cystic lesions were identified surrounding and infiltrating the T11 vertebra. Surgical debulking of the T11 vertebra was performed until no residual cystic components remained; haemorrhagic elements were also observed. The resulting cavity was filled with a synthetic bone graft. Posterior spinal instrumentation was carried out from T9 to L1, and a mesh cage was inserted between T10 and T12 to facilitate spinal fusion. The total operative time was two hours and 54 minutes, with an estimated blood loss of 500 cc.

**Figure 1:**
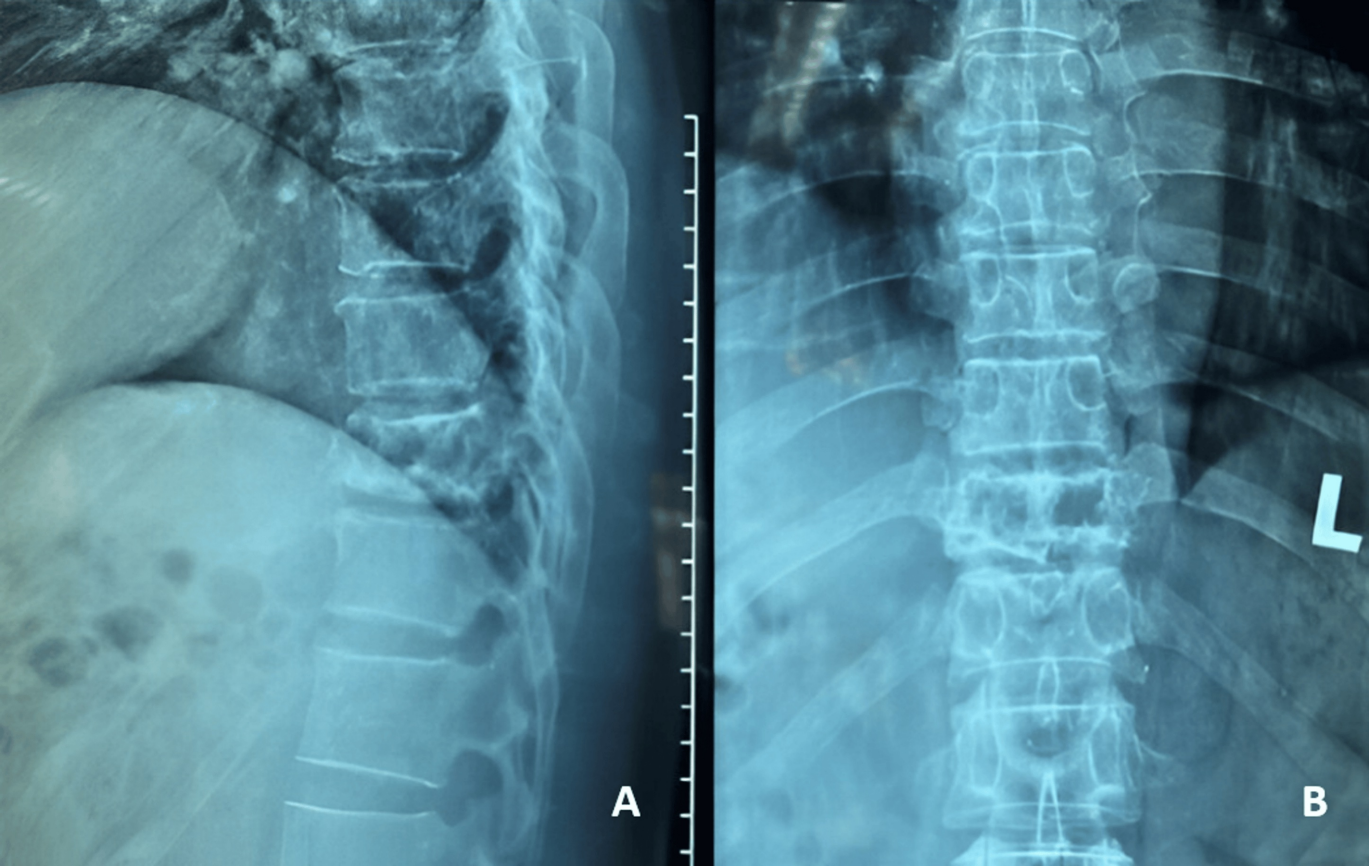
Preoperative plain radiographs of thoracic spine. A: Lateral view. B: AP view.

**Figure 2:**
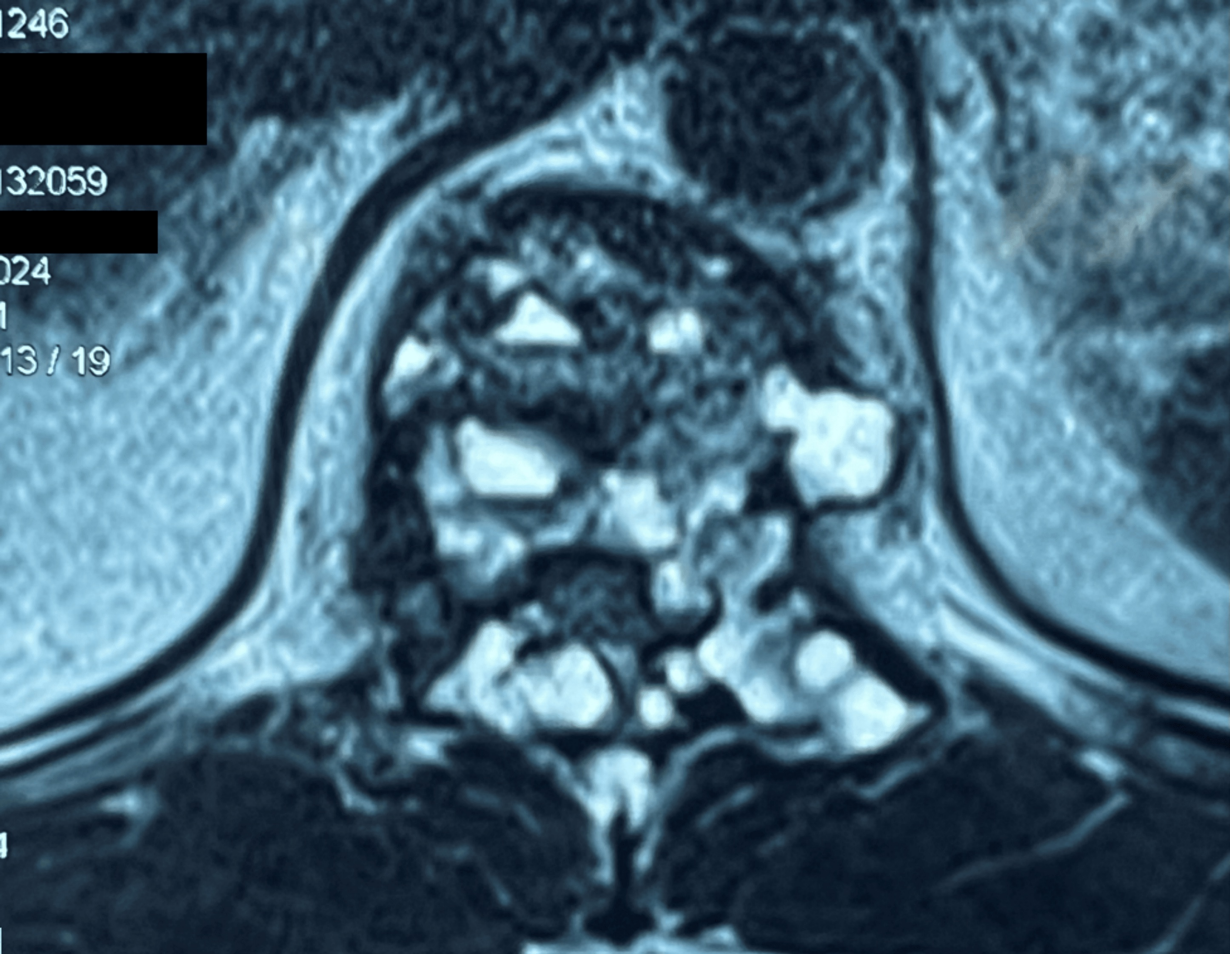
MRI scan of thoracolumbar spine with contrast, T2-weighted (axial section) showing characteristic findings of aneurysmal bone cyst: multiloculated lesion at T11 vertebra with multiple fluid-fluid levels.

**Figure 3:**
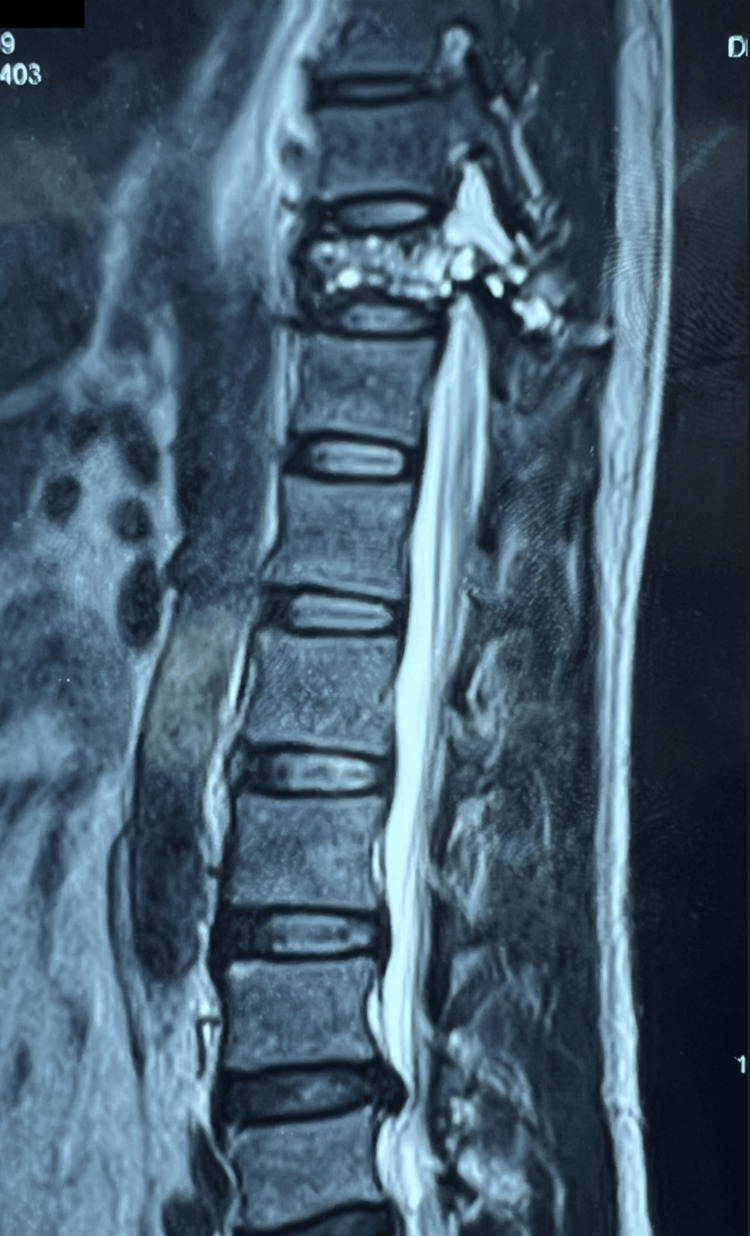
MRI scan of thoracolumbar spine with contrast, T2-weighted (sagittal section) showing characteristic findings of aneurysmal bone cyst: multiloculated lesion at T11 vertebra with multiple fluid-fluid levels.

**Figure 4:**
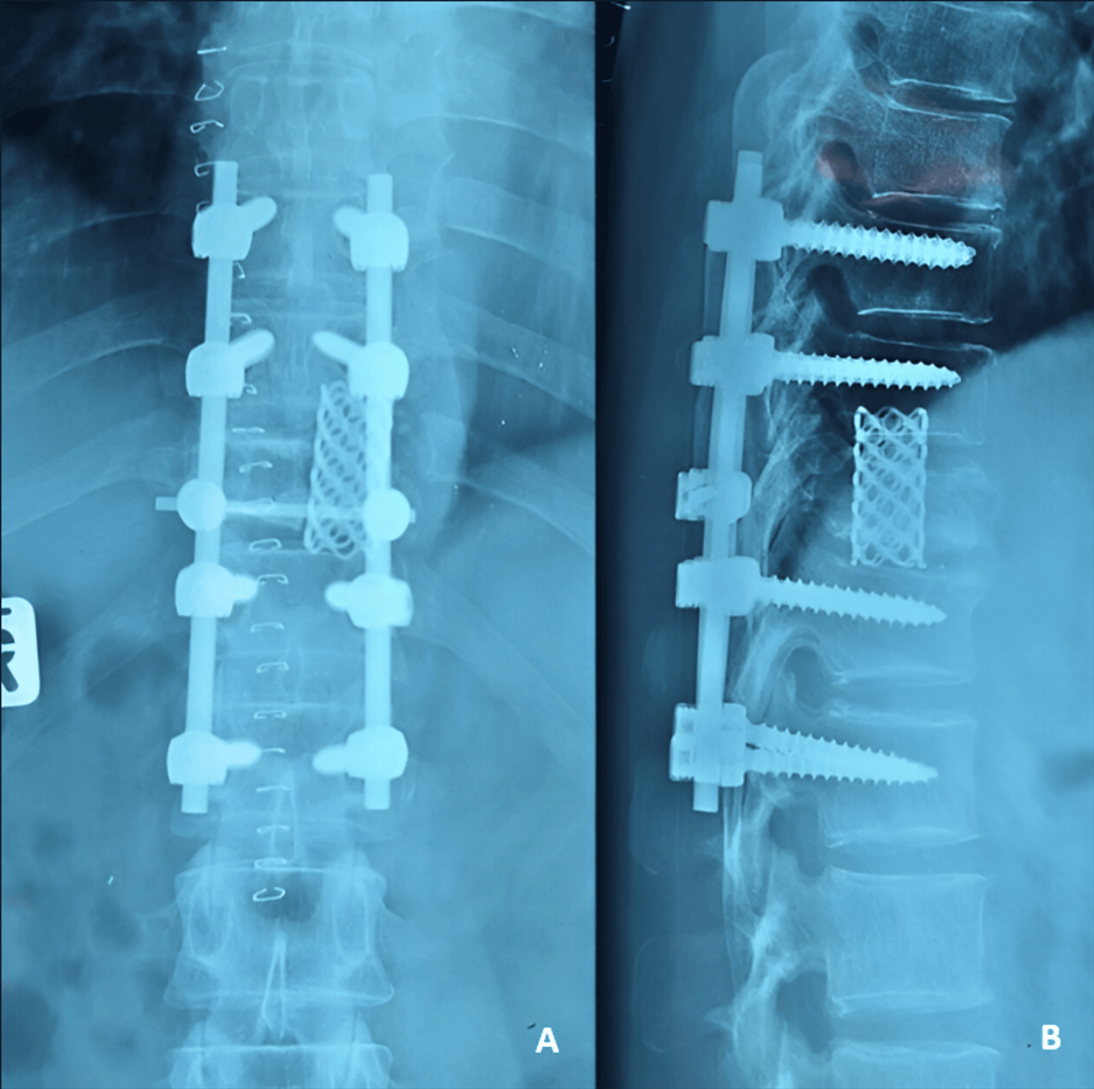
Postoperative thoracolumbar plain radiograph showed pedicle screw fixation done from T9 to L1 with mesh cage at level T11. A: AP view. B: Lateral view.

**Figure 5:**
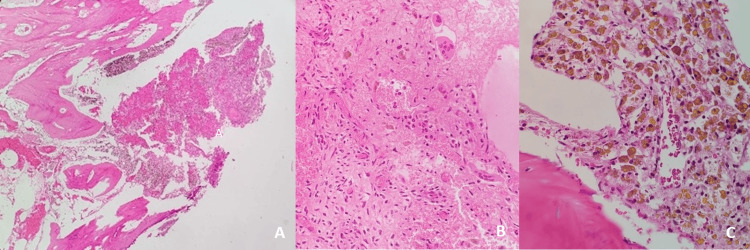
Histopathology. A: Blood-filled cystic space with fibroblasts, 40× magnification. B: Multinucleated giant cells and hemosiderin-laden macrophages, 400× magnification. C: Multinucleated giant cells and hemosiderin-laden macrophages, 200× magnification.

**Figure 6:**
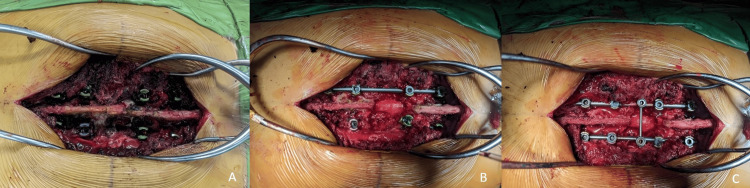
Intraoperative images. A: Four pairs of screws inserted into pedicles. B and C: Hyperaemic spinal cord seen post-decompression and tumour debulking.

Postoperative histopathological examination (HPE) confirmed the diagnosis of an ABC, and the patient experienced significant pain reduction. At the six-month follow-up, he remained symptom-free with intact neurological function (Figures 1-6).

## Discussion

Primary ABCs are uncommon, benign cystic bone tumours that predominantly occur in children and adolescents. They can exhibit locally aggressive behaviour. ABCs most frequently occur in individuals aged 10 to 20 years, with a slight predominance in females. The average age at diagnosis is around 13 years, and 75% to 90% of cases are identified before the age of 20. ABCs account for about 2% of all benign bone tumours, with an incidence of 0.14 per 100,000 individuals [2].

The lesion typically begins in the pedicles and subsequently extends into the vertebral body. However, some researchers suggest that it originates in the posterior elements, progressing to involve the pedicle before reaching the vertebral body. ABC is thought to arise from a pre-existing lesion due to a secondary vascular phenomenon. This event leads to the formation of a periosteal or intraosseous arteriovenous malformation, which causes erosion of the bone trabeculae and the formation of a cystic cavity. Alternatively, molecular studies have identified recurrent translocations involving the USP6 gene, suggesting a neoplastic origin. This genetic alteration is thought to drive osteoclast activation and bone resorption, aligning with the aggressive behaviour observed in some ABCs.

Pain is the most common symptom. ABCs are generally diagnosed due to pain, a palpable mass, or a pathologic fracture due to the extensive bone weakening caused by the cyst. In spinal ABCs, patients may manifest as sensory deficits, motor weakness, paravertebral mass, kyphoscoliosis, radiculopathy or myelopathy, warranting prompt intervention. 

Diagnosing ABCs involves a combination of clinical assessment, imaging studies, and histological analysis. X-rays may show vertebral ballooning, the “winking owl” sign or compression fracture. A CT scan reveals the precise extent of the lesion and the fluid-filled cavities within the bone. On MRI, ABCs are characteristically seen as multiloculated cysts with fluid-fluid level, reflecting blood at different stages of evolution. The internal septations typically enhance with contrast administration. MRI helps evaluate spinal cord compression and paravertebral extension.

MRI and CT scans are invaluable for assessing the full extent of the lesion and its relationship with surrounding tissues, providing a detailed roadmap for surgical planning. Histologically, ABCs are marked by blood-filled cavities separated by fibrous septa, containing multinucleated giant cells and reactive bone formation. MRI, often combined with histologic confirmation, is the preferred method for diagnosing ABCs, as telangiectatic osteosarcoma (TOS) has similar imaging characteristics and should be ruled out.

Differential diagnosis includes giant cell tumour, haemangioma, fibrous dysplasia, osteosarcoma, osteoblastoma, and metastatic lesions. ABCs typically exhibit a sponge-like appearance, consisting of multiloculated, blood-filled spaces separated by thin fibrous septa. These septa are composed of fibroblasts, myofibroblasts, multinucleated osteoclast-like giant cells, hemosiderin deposits, blood vessels, and areas of osteoid and woven bone.

Treatment modalities

The primary goal of ABC treatment is to eradicate the lesion, prevent recurrence, and restore normal bone function while minimising complications such as fractures and growth disturbances.

Current treatment strategies for ABC include percutaneous intralesional sclerotherapy, for example, with polidocanol, bone marrow injections, cryotherapy, decompression, curopsy, curettage with or without filling and/or chemical adjuvants and/or high-speed burring, systemic therapies, radiofrequency ablation, embolisation, percutaneous injectable bone substitutes, wide resections, and a wide range of osteosyntheses if necessary.

Surgical Curettage and Grafting

This is the most widely used treatment, involving the removal of the cystic tissue followed by bone grafting to fill the defect. Despite being effective, recurrence rates can be as high as 20-30%. A retrospective study revealed that curettage and impaction of bone graft is an efficient and reproducible treatment, and it showed that no local recurrence, infection, or pathological fractures occurred during the two-year postoperative period [3].

En Bloc Resection

Reserved for aggressive or recurrent lesions, this method involves the complete surgical removal of the affected bone segment. While this reduces recurrence, it may lead to significant functional impairment depending on the bone involved.

Minimally Invasive Techniques

Percutaneous sclerotherapy, where agents like polidocanol or doxycycline are injected into the cyst, and radiofrequency ablation have emerged as effective alternatives with lower morbidity. These techniques have demonstrated promising results in terms of reducing recurrence and facilitating quicker recovery.

Adjuvant Therapies

The application of adjuvants, such as phenol, liquid nitrogen, or bone cement, following curettage can help minimise recurrence. Denosumab, a monoclonal antibody targeting receptor activator of nuclear kappa B ligand (RANKL), has shown efficacy in treating recurrent or inoperable ABCs by inhibiting osteoclast activity and promoting bone healing.

Patients without neurological symptoms, whose only complaint is pain, may be managed with non-surgical treatments such as selective arterial embolisation, intralesional injections of calcitonin and methylprednisolone, or vertebroplasty. Embolisation promotes regression of the soft tissue component and induces sclerosis and ossification. Calcitonin injections encourage cancellous bone formation and inhibit osteoclastic activity, while methylprednisolone reduces fibroblastic action and angiogenesis.

In contrast, patients with neurological deficits require surgical intervention. Simple curettage, with or without bone grafting, is not recommended in spinal cases due to a reported recurrence rate of around 19% within two years. The preferred treatment is complete surgical excision, which offers excellent outcomes. Complete removal must include the entire cyst wall and all abnormal tissue, including the spongy areas and bone surfaces covered with fragile, hypervascular membranes. Intraoperative bleeding originates from the sinusoidal lining of the thin-walled vascular spaces and can be challenging to control until the entire lining is excised. For extensive lesions or those causing spinal instability, stabilisation is required. Radiation therapy alone offers little benefit and carries the risk of malignant transformation. However, adjuvant radiation may be considered in select cases, such as inoperable lesions, highly vascular tumours, aggressive recurrences, or patients who are poor surgical candidates.

External beam radiotherapy may be employed as primary treatment for ABC. However, complications associated with radiotherapy include late-onset axial deformities (particularly in spinal lesions in adolescents), growth plate arrest, myelopathy, and the risk of radiation-induced sarcomas. While late axial deformities can also occur after curettage, they are significantly more common following radiotherapy. A major concern is the development of post-radiation sarcoma, transforming a benign condition with no malignant potential into a fatal outcome.

HPE discussion

ABC: Characterised by blood-filled cystic cavities divided by fibrous septa that contain multinucleated giant cells, fibroblasts, and areas of reactive bone formation. No malignant cells are present.

Macroscopic findings: An ABC primarily consists of blood-filled cystic spaces enclosed by a thin layer of reactive bone. In some cases, solid components may also be observed.

Microscopic findings: ABCs are characterised histologically by both solid areas and cystic spaces filled with blood, lacking any epithelial or endothelial lining and separated by cellular septa. Three main histological components are typically seen: (1) a cellular component containing osteoclast-like multinucleated giant cells with high expression of receptor activator of nuclear kappa B (RANK) and neoplastic stromal mononuclear and myofibroblastic cells expressing high levels of RANKL, (2) a fibrillar component that composed of collagenous extracellular matrix, and (3) an osteoid component made up of unmineralized bone matrix deposited by osteoblasts. Mitoses are often prominent; however, cytologic atypia is absent, and necrosis is rarely observed [4].

TOS: Features blood-filled spaces like ABCs, but with the addition of malignant osteoblastic cells. In contrast to an ABC, TOS features a delicate, lacelike osteoid matrix. Also, the stroma between the dilated vascular spaces often contains malignant cells, showing highly atypical, pleomorphic cells, characteristic of osteosarcoma.

Macroscopic findings: A haemorrhagic multicystic lesion containing blood clots is observed. TOSs are often described as "a bag of blood," with about 90% of the lesion consisting of cystic components before treatment. Typically, a fleshy sarcomatous component is absent. Instead, the lesion consists of a mix of large cystic spaces and spongy areas, the latter characterised by tissue with a honeycomb-like pattern formed by numerous small cysts measuring up to several millimetres. While the lesion typically has well-defined borders, it frequently exhibits invasive behaviour, including marked irregular erosion of the cortex, complete loss of cortical integrity, and extension into adjacent soft tissues.

Microscopic findings: Histologically, TOS features blood-filled or empty cystic spaces similar to those seen in ABC. The septa contain pleomorphic cells with nuclear hyperchromasia, as well as osteoclast-like giant cells. Osteoid production is typically focal. Key microscopic features distinguishing TOS from ABC include thickened, nodular septa and the presence of malignant cells showing nuclear pleomorphism and cellular atypia [5].

Prognosis and follow-up

The prognosis for patients with ABCs is generally favourable, with most lesions responding well to treatment. However, the risk of recurrence remains a significant concern, necessitating careful follow-up. Recurrences are most likely to occur within the first two years post-treatment, emphasising the need for regular imaging studies to detect any signs of relapse or complications early. Long-term outcomes are generally positive, especially with advancements in minimally invasive techniques and adjuvant therapies, which have improved the management and reduced the morbidity associated with traditional surgical methods.

## Conclusions

ABC is an uncommon bone tumour found in the spine. Despite its benign classification, ABCs can pose significant clinical challenges due to their locally aggressive nature and potential for recurrence. Surgical decompression is necessary in cases of root or spinal cord compression. Stabilisation may be necessary, as posterior elements of the vertebral body are often involved, causing instability. Surgical curettage, bone grafting, and stabilisation with instrumentation can lead to favourable clinical outcomes, as demonstrated in this case.

Advances in the understanding of their molecular basis have provided insights into their neoplastic nature, guiding the development of targeted therapies. A combination of surgical and minimally invasive techniques, supplemented by adjuvant therapies, offers effective management options. Continued research and clinical trials are essential to refine these approaches further, aiming to enhance patient outcomes and minimise recurrence rates.
